# Color Filtering Localization for Three-Dimensional Underwater Acoustic Sensor Networks

**DOI:** 10.3390/s150306009

**Published:** 2015-03-12

**Authors:** Zhihua Liu, Han Gao, Wuling Wang, Shuai Chang, Jiaxing Chen

**Affiliations:** 1 College of Information Technology, Hebei Normal University, Shijiazhuang 050024, China; E-Mails: liuzhihua@mail.hebtu.edu.cn (Z.L.); wwling2001@163.com (W.W.); 2 College of Mathematics and Information Science, Hebei Normal University, Shijiazhuang 050024, China; E-Mails: gaohan19920113@163.com (H.G.); changshuai1101@163.com (S.C.)

**Keywords:** underwater acoustic sensor networks (UASNs), self-localization, color filtering, Red Green Blue (RGB), hierarchical structure

## Abstract

Accurate localization of mobile nodes has been an important and fundamental problem in underwater acoustic sensor networks (UASNs). The detection information returned from a mobile node is meaningful only if its location is known. In this paper, we propose two localization algorithms based on color filtering technology called PCFL and ACFL. PCFL and ACFL aim at collaboratively accomplishing accurate localization of underwater mobile nodes with minimum energy expenditure. They both adopt the overlapping signal region of task anchors which can communicate with the mobile node directly as the current sampling area. PCFL employs the projected distances between each of the task projections and the mobile node, while ACFL adopts the direct distance between each of the task anchors and the mobile node. The proportion factor of distance is also proposed to weight the RGB values. By comparing the nearness degrees of the RGB sequences between the samples and the mobile node, samples can be filtered out. The normalized nearness degrees are considered as the weighted standards to calculate the coordinates of the mobile nodes. The simulation results show that the proposed methods have excellent localization performance and can localize the mobile node in a timely way. The average localization error of PCFL is decreased by about 30.4% compared to the AFLA method.

## 1. Introduction

Underwater acoustic sensor networks (UASNs) are composed of thousands of micro-sensors which are capable of sensing, operating, self-organizing and communicating acoustically to monitor the underwater environment [1,2,3]. In UASNs, technology provides new capacity for marine resources exploration, pollution detection and aided navigation [4]. Nowadays, research on communication technology [3], architectures and protocols [3], localization and tracking algorithms [5], as well as network security [6] have been applied to various fields. As for a variety of applications in UASNs, the acquisition of information is meaningful only when the location of the sensors is known. Underwater acoustic mobile localization (UABL) technology has been widely studied since it is a primary tool used in topology control, coverage control and routing decision in UASNs [7,8]. UABL typically employs a cluster of anchors whose locations could be obtained in advance and a set of mobile nodes whose locations are to be determined. Based on the information received from the anchors, mobile nodes perform self-localization which belongs to the classification of distributed localization techniques [2]. The model where each mobile node is equipped with a pressure sensor is motivated by reference [8] in which an anchor-free localization algorithm called AFLA was presented. AFLA was designed for active-restricted underwater sensor networks and made use of the relationships between adjacent nodes. Comprehensive surveys of these UABL schemes are presented in references [2,7]. As global positioning system (GPS) signals are greatly weakened underwater, UABL algorithms often use range-based methods to estimate distance, *i.e.*, time of arrival (TOA) [9], angle of arrival (AOA) [10] and time difference of arrival (TDOA) [11].

In this article, a projection-color filtering localization algorithm called PCFL and an anchor-color filtering localization algorithm called ACFL are put forward. They both aim at cooperatively accomplishing precise localization for underwater mobile nodes with minimum power wastage. In the first place, the existing network construction is reconstituted as a hierarchical structure and the localization issue is converted into a geometry problem. Secondly, based on the task anchors which can communicate with the mobile node directly, task-rings are obtained considering the task projections (*i.e.*, projections of the task anchors) as centers, and samples are randomly selected in the overlapping area of the task-rings. Later, the Red Green Blue (RGB) sequences for both the mobile nodes and the samples are computed based on the projection distances. Different from the existing CDL algorithms utilizing the DV-hop measurement [12], PCFL uses the AOA measurement and the initial RGB values are given to the task projections, while for ACFL, the initial RGB values are given to the task anchors. Last, the nearness degree is defined to filter samples, and at the same time, it is stored as a weight.

Our localization strategy is motivated by the existing CDL algorithms [12], such as making use of the locations and RGB values, from the anchors, to assist the mobile node to compute its RGB value and exploiting the changes of colors with distances to localize the mobile node. The CDL aims to represent a location with a color instead of a coordinate for cutting down the computation and communication costs. With a color to represent a location, two kinds of data are fused into a color: (1) each anchor’s location and (2) the distance between the mobile node and each anchor. Also we are motivated by the main idea of hierarchical structure [13] which is that each layer value represents the Z axis coordinate of each anchor in the three-dimensional underwater environment. Since the anchors are distributed by layers, the three dimensional distance measurements can be converted to two-dimensional ones by using the distance between the anchors’ projection and the anchors.

The major contributions of this work are as follows: (a) based on a detailed theoretical analysis PCFL and ACFL are proposed for three-dimensional underwater environments; (b) the proposed algorithms adopt the overlapping signal region of task anchors which can reduce uncertainty with fewer anchors than used in other approaches; (c) it is more efficient by using the adapted hierarchical structure and the projection model; (d) as the proportion factor of distance is proposed to weight the RGB values, PCFL and ACFL can provide more accurate RGB values; (e) specifically, the normalized nearness together with the calculation of the coordinates significantly improves the whole computational efficiency.

The rest of this paper is arranged as follows: in Section 2, we survey some of the existing range-free localization techniques in UASNs. In Section 3, the PCFL and ACFL algorithms are put forward and their performance is verified, where we deduce the task-rings sampling method, compute the nearness degree threshold for filtering samples and locate the weighted mobile nodes. Simulations of the performance of PCFL and ACFL are evaluated in Section 4, and in the end conclusions are given in Section 5. The key notations used in this paper are summarized in Table 1.

**Table 1:** List of key notations.

Notation	Explanation
R	Communication range
UN	A 3D UASNs
p	Number of anchors
q	Number of mobile nodes
$n_{j}(j=1,2,...p+q)$	Anchors or mobile nodes
A	Set of anchors
M	Set of mobile nodes
$\alpha_{ij}$	Smaller angle of acoustic signal from the anchors $n_{j}$ received by the mobile node $n_{i}$
$d(n_{i},n_{j})$	Euclidean distance between the node $n_{i}$ and the node $n_{j}$
$A_{i}^{t}$	The set of task anchors corresponding to the mobile node $n_{i}$
$p^{t}$	Number of task anchors in $A_{i}^{t}$
$B_{i}^{t}$	The set of task projections for $n_{j}\in A_{i}^{t}$
$RGB_{j}^{pt}$	RGB sequences for the projections in $B_{i}^{t}$ at time instant t
$RGB_{j}^{at}$	RGB sequences for the anchors in $A_{i}^{t}$ at time instant t
$C_{j}^{t}$	Task-ring for task projection ${n^{\prime }}_{j}\in B_{i}^{t}$
$k_{ij}^{t}$	Depth difference between the task anchor $n_{j}$ and the mobile node $n_{i}$
${n^{\prime }}_{j}$	Projection of the task anchor $n_{j}$
$p_{ij}^{t}$	Distance between the mobile node $n_{i}$ and the task anchor’s projection ${n^{\prime }}_{j}$
$S_{j}^{t}$	The set of the sampling area
$\lambda_{ij}^{t}$	Proportion factor of distances weights
$s_{k}$	The *k*th sample
$\mu_{s_{k}M}^{t}$	Nearness degree between the mobile node $n_{i}$ and the sample $s_{k}$
${\tilde{S}}_{i}^{t}$	The filtered samples set
$\mu^{t}$	Threshold for nearness degree at time instant t
$m^{t}$	Number of the filtered samples at time instant t

## 2. Background

### 2.1. Related Studies

Some researchers have studied range-free algorithms, which do not need to estimate the distance precisely [14,15,16]. Instead, sensors can localize themselves by taking advantage of signal connectivity, delay time, hop-distance and angle information. Range-free schemes are always more economic and simpler than the range-based ones, but less accurate. 

Color-theory based dynamic localization (CDL) [12], FRORF [15] and DV-hop algorithms [14] are range-free methods which do not acquire distance information, but they always have coarser performance. CDL calculates RGB sequences based on DV-hop for both samples and mobile nodes. It converts RGB to Hue, Saturation, Value (HSV) for mobile nodes and samples using the traditional convert algorithm. Color theory indicates that the information of RGB and HSV fuses different red, green and blue data. According to the color theory, only the V (value) of HSV changes in proportion to the distances between anchors and mobile nodes. After the new HSV was obtained, another RGB data of the nodes is acquired by the HSV to RGB method. Then CDL filters the nearest sample by searching for the most similar RGB sequence to the mobile node, and identifies it as the location of the mobile node. Although CDL has better location performance in various terrestrial networks, it’s not suitable for localization in UASNs due to the inconsistent distribution of nodes and the weak association between signal hops and actual distances. Liu *et al.* proposed a local sampling and filtering color dynamic localization (LSF-CDL) algorithm [17]. Using the collected signals, LSF-CDL adopts the overlapping signal region of anchors which were able to communicate with the mobile node directly as the new local sampling area. The proportion factor of distance was also used to weight the average hop distance which optimized the calculation of hop distance in CDL. By comparing the RGB difference sequences, samples could be filtered out. The FRORF method represented overlapping rings as fuzzy sets to isolate a region where the node was most likely located [15]. The localization accuracy was improved under different number of anchors and degrees of radio propagation irregularity. 

In the color-theory based localization schemes, a location database is established in the server for building geographic locations as a function of RGB values. The DV-Hop technology is employed in measuring the distances between mobile nodes and anchors. Based on the RGBtoHSV algorithm [12], the mobile node converts the RGB values received from the anchors into the corresponding HSV values [12,17]. According to the color theory, the increase of propagation distances only fades the lightness of color, which is the V in HSV of an anchor. Namely, V decreases proportionately to the distance between the mobile node to the anchor. After the mobile node gets the new HSV values, it adjusts these HSV values into RGB values based on another conversion algorithm called HSVtoRGB [12]. Then the mobile node averages these converted RGB values as its own RGB values corresponding to the anchors. Further, the server can find out the most probable location for the mobile node after receiving its RGB values and searching the location database. Since CDL is centralized and easy to implement, for applications that need centralized user data collection and user activity monitoring, such as community health-care systems and hospital monitoring systems, CDL is an ideal scheme to be used.

Compared to terrestrial nodes which can keep their location unchanged after arrangement, underwater nodes are prone to be influenced by tides, ocean currents and other factors leading to their locations being unfixed [1]. Due to the influence of node mobility, multipath fading and shadow, long time delay, the variation of sound velocity and asymmetry factor, there are more challenges for the UABL algorithms [18]. The autonomous underwater vehicle (AUV)-aid method employed a large number of sensors and one AUV to balance the algorithm’s performance and cost. The AUV was used for localization and carrying messages of disconnected sensors or time-critical information [19]. The anchor-based method relied on the TDOA locally measured by a sensor to detect range differences from the sensor to four anchors [20]. The AFLA method was a self-localization algorithm designed for anchor-free UASNs [8]. However, none of the schemes mentioned above provide sufficient accuracy. In order to improve the localization performance, we propose here two novel algorithms: PCFL and ACFL, and aim at projecting the locations of task anchors to the mobile node’s plane which converts the UABL problem from a 3D to a 2D one. 

### 2.2. Network Model

**Figure 1:**
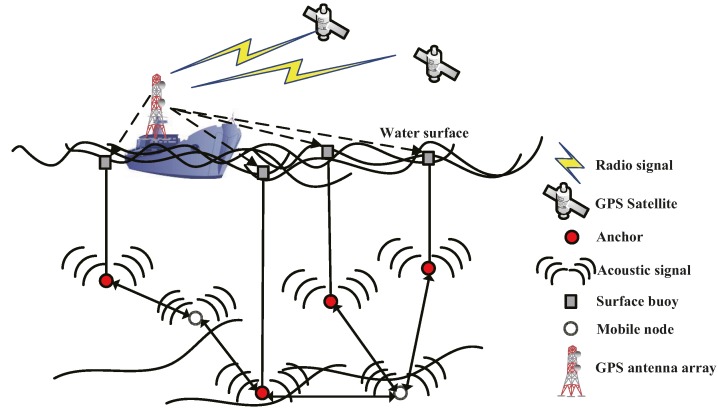
Underwater acoustic sensor networks structure.

There is a typical UASNs model as shown in Figure 1. There are three types of nodes in the model: mobile nodes, anchors and surface buoys [21]. Surface buoys are deployed on the water surface and often equipped with GPS to get their absolute locations with the help of GPS antenna arrays and GPS satellites. Anchors and mobile nodes communicate with each other using acoustic signals. Each anchor is vertically connected with one surface buoy by a cable. Anchors can obtain the two-dimensional coordinates with the help of the surface buoys. The main role of the anchors is helping the mobile nodes to finish self-localization.

### 2.3. Problem Formulation

With the purpose of developing an accurate and high-performance UABL scheme used in the complex underwater environments, the assumptions are made as below: 

Both the anchors and the mobile nodes have the same communication range *R* and are equipped with arrays of wireless antennas in order to communicate with each other by acoustic signals which are used to transmit the depth information, the anchors’ coordinates and the measured AOA values. 

The mobile node transmits acoustic signals regularly. Once the acoustic signal is received, each anchor replies a message including its own three dimensional coordinates and its depth information.

When the information exchange is completed, the mobile node has sufficient information to localize itself. The information refers to locations and the depth information of all task anchors and the AOA measurement from the task anchors estimated by the mobile node. The depth information is measured by the pressure sensors with which all the nodes are equipped.

In UASNs, it is difficult to achieve time synchronization precisely as a result of the characteristics of acoustic signal propagation. While by employing the AOA values of the acoustic signals from the anchors, synchronous request between the nodes is not so necessary, so the PCFL and ACFL techniques advanced here have the advantage in synchronization.

Let’s consider a 3D UASNs *UN* with p anchors and q mobile nodes and express the location of each node as:

(1) $$n_{i}=(x_{i},y_{i},z_{i}),n_{i}\in UN,i=1,2,...p+q$$

We assume that the locations of p anchors in $A=\{n_{1},n_{2},...n_{p}\}$ are known but the locations of the other *q* mobile nodes in $M=\{n_{p+1},n_{p+2},...n_{p+q}\}$ are undetermined and to be localized, UN=A∪M. In our localization methods, the only obtainable information is the smaller angle of acoustic signal $\alpha_{ij}$ from the anchors $n_{j}(j=1,2,...p)$ received by the mobile node $n_{i}(i=p+1,...p+q)$ based on the AOA measurement and the depth information obtained by the corresponding pressure sensors. The Euclidean distance between the node $n_{i}$ and the node $n_{j}$ can be calculated as:

(2) $$d(n_{i},n_{j})=\sqrt{{\left(x_{i}-x_{j}\right)}^{2}+{\left(y_{i}-y_{j}\right)}^{2}+{\left(z_{i}-z_{j}\right)}^{2}},(n_{i}\in UN,n_{j}\in UN)$$

At time instant t, the set of task anchors corresponding to the mobile node $n_{i}$ can be expressed as

(3) $$A_{i}^{t}=\{n_{j}|d(n_{i},n_{j})\leq R,i=p+1...p+q,j=1,2,...p\}\subset A$$

Let $p^{t}$ denote the number of task anchors in $A_{i}^{t}$ and it can be seen that:

(4) $$\left|A_{i}^{t}\right|=p^{t}\leq p$$

As all the nodes have the same communication range R, at time instant t, the mobile node $n_{i}(i=p+1,...p+q)$ can exchange information with anchor $n_{j}$ directly if and only if $n_{j}\in A_{i}^{t}$. The set of task projections $B_{i}^{t}$ for $n_{j}\in A_{i}^{t}$ is defined as:

(5) $$B_{i}^{t}=\{n_{j}^{\prime }|d(n_{i},n_{j})\leq R,{x^{\prime }}_{j}=x_{j},{y^{\prime }}_{j}=y_{j},{z^{\prime }}_{j}=z_{j}\}\left|B_{i}^{t}\right|=p^{t}\leq p$$

where ${n^{\prime }}_{j}=({x^{\prime }}_{j},{y^{\prime }}_{j},{z^{\prime }}_{j})$. RGB sequences for the projections in $B_{i}^{t}$ and RGB sequences for the anchors in $A_{i}^{t}$ at time instant t are randomly assigned numerical values in the range of [0,1] as stated in the reference [12] and respectively are defined as:

(6) $$RGB_{j}^{pt}:\{R_{j}^{pt},G_{j}^{pt},B_{j}^{pt}\},({n^{\prime }}_{j}\in B_{i}^{t})\text{ and }RGB_{j}^{at}:\{R_{j}^{at},G_{j}^{at},B_{j}^{at}\},(n_{j}\in A_{i}^{t})$$

The task-ring for task projection ${n^{\prime }}_{j}\in B_{i}^{t}$ is defined as:

(7) $$C_{j}^{t}=\{\left(x,y,z\right)|(x-{x^{\prime }}_{j})+(y-{y^{\prime }}_{j})\leq (R^{2}-{\left(k_{ij}^{t}\right)}^{2}),z={z^{\prime }}_{j}\}$$

where $k_{ij}^{t}(j=1,...p^{t},i=p+1,...q)$ is the depth difference between the task anchor $n_{j}$ and the mobile node $n_{i}$ which can be calculated using the information from their equipped pressure sensors. Note that the minimum communication angle between the task anchor $n_{j}$ and the mobile node $n_{i}$ at time instant t named as $\alpha_{ij}^{t}(j=1,...p^{t},i=p+1,...q)$ can be measured by the mobile node $n_{i}$, then the localization issue can be described as follows:

(8) $$\begin{array}{l} Estimate\;n_{i}\\ subject\;to\;n_{j}(n_{j}\in A_{i}^{t})({n^{\prime }}_{j}({n^{\prime }}_{j}\in B_{i}^{t}));\alpha_{ij}^{t};k_{ij}^{t};(i=p+1,...q) \end{array}$$

## 3. Algorithm Design

In this section, we present our color filtering localization algorithms, which we call projection-color filtering localization (PCFL) and anchor-color filtering localization (ACFL). Firstly, we put forward the three dimensional hierarchical structure according to the depth of the anchors and the mobile nodes. Then we introduce the design details of PCFL and ACFL methods. In the end, we show the analysis of feasibility for them. PCFL and ACFL are both based on the color theory. The distances between nodes are calculated by the projection method, while CDL calculates the distance based on DV-hop method. 

### 3.1. Hierarchical Structure Model

In UASNs, the most important characteristics are node mobility, multipath propagation loss, time uncertainty, and low communication rate. In this paper, our two localization methods are based on hierarchical structure model. 

**Figure 2:**
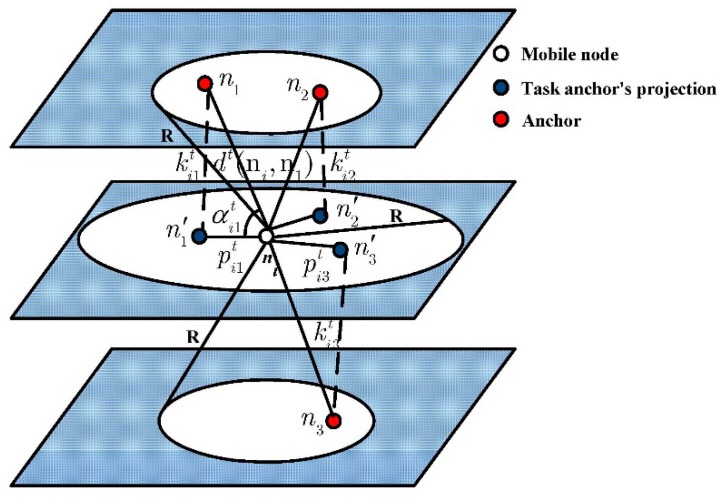
Hierarchical and projection structure model.

Figure 2 shows a hierarchical structure and projection model. Using the depth information of the mobile node, three task anchors $n_{1}$, $n_{2}$ and $n_{3}$ will be projected to three positions ${n^{\prime }}_{1}$, ${n^{\prime }}_{2}$ and ${n^{\prime }}_{3}$ in the plane where the mobile node locates, respectively.

Note that the minimum communication angle $\alpha_{ij}^{t}\in [0,\frac{\pi }{2}](j=1,2,3)$ can be obtained by the mobile node $n_{i}$ based on the AOA measurement, and the depth difference $k_{ij}^{t}(j=1,2,3)$ can be measured by the equipped pressure sensors. When the anchor $n_{j}$ and the mobile node $n_{i}$ are with the same depth, $\alpha_{ij}^{t}=0$, $k_{ij}^{t}=0$. When $\alpha_{ij}^{t}=\frac{\pi }{2}$, the value of the minimum communication angle between $n_{j}$ and $n_{i}$ will be maximum. So at time instant t the geographic distance between the anchor $n_{j}$ and the mobile node $n_{i}$ can be calculated as:

(9) $$d^{t}(n_{i},n_{j})=k_{ij}^{t}/\text{sin}\alpha_{ij}^{t}$$

And at time instant t, the distance $p_{ij}^{t}$ between the mobile node $n_{i}$ and the task anchor’s projection ${n^{\prime }}_{j}$ can be calculated as:

(10) $$p_{ij}^{t}=k_{ij}^{t}/\text{tan}\alpha_{ij}^{t}$$

### 3.2. PCFL and ACFL

The PCFL algorithm and the ACFL algorithm can be both divided into three steps: (a) determination of the sampling area; (b) RGB values calculation; and (c) filtering and weighted evaluation. 

(a) In the first step, we use the task-rings sampling method to determine the sampling area. Here every task-ring is expressed as the signal range interface for every corresponding task anchor, as previously defined in Equation (7). Then, the sampling area constraining the location of the mobile node is the intersection region of these task-rings corresponding to each task projection.

**Figure 3:**
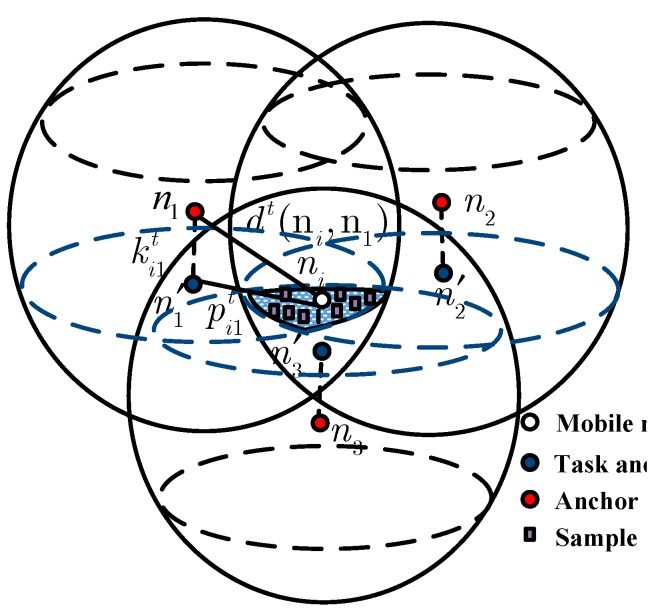
The sampling area.

For instance, the shadow region in Figure 3 represents the sampling area which is derived from the task-rings of task projections ${n^{\prime }}_{1}$, ${n^{\prime }}_{2}$ and ${n^{\prime }}_{3}$ with respect to task anchors $n_{1}$, $n_{2}$ and $n_{3}$, respectively. The task-rings are represented as the blue dotted circles. As each task anchor can receive the signal from the mobile node, it can be inferred that the mobile node’s location is inside its task-ring. The following Theorem 1 will prove this. The following restricted conditions based on the task-rings sampling method will confine the samples shown as the purple squares inside the shadow region in Figure 3:

(11) $$\sqrt{{\left(x-{x^{\prime }}_{1}\right)}^{2}+{\left(y-{y^{\prime }}_{1}\right)}^{2}}\leq \sqrt{\left(R^{2}-{\left(k_{\text{i1}}^{t}\right)}^{2}\right)},z={z^{\prime }}_{1}=z_{i}\;\sqrt{{\left(x-{x^{\prime }}_{2}\right)}^{2}+{\left(y-{y^{\prime }}_{2}\right)}^{2}}\leq \sqrt{\left(R^{2}-{\left(k_{i2}^{t}\right)}^{2}\right)},z={z^{\prime }}_{2}=z_{i}\;\sqrt{{\left(x-{x^{\prime }}_{3}\right)}^{2}+{\left(y-{y^{\prime }}_{3}\right)}^{2}}\leq \sqrt{\left(R^{2}-{\left(k_{i3}^{t}\right)}^{2}\right)},z={z^{\prime }}_{3}=z_{i}$$

where (*x*, *y*, *z*) denotes the coordinates of the sample. Normally, the set of the sampling area $S_{i}^{t}$ is defined as:

(12) $$S_{i}^{t}=\{\left(x,y,z\right)|\forall {n^{\prime }}_{j}\in B_{i}^{t},\sqrt{\left(x-{x^{\prime }}_{j}\right)+{\left(y-{y^{\prime }}_{j}\right)}^{2}}\leq \sqrt{\left(R^{2}-{\left(k_{ij}^{t}\right)}^{2}\right)},z=z_{i}\}$$

(b) The process of RGB values calculation can be divided into two stages. The first stage is the RGB sequences calculation for the mobile nodes, denoted by $RGB_{i}^{Mt}:\{R_{i}^{Mt},G_{i}^{Mt},B_{i}^{Mt}\},(n_{i}\in M)$. After arranging all the RGB values $RGB_{j}^{pt}:\{R_{j}^{pt},G_{j}^{pt},B_{j}^{pt}\},({n^{\prime }}_{j}\in B_{i}^{t})$ for task anchors’ projections (PCFL) or arranging all RGB values $RGB_{j}^{at}:\{R_{j}^{at},G_{j}^{at},B_{j}^{at}\},(n_{j}\in A_{i}^{t})$ for task anchors (ACFL), the mobile node converts $RGB_{j}^{pt}$ or $RGB_{j}^{at}$ into Hue, Saturation, and Value (HSV) [12] at time instant t.

(13) $$H_{j}^{kt}S_{j}^{kt}V_{j}^{kt}=(RGB\text{ to }HSV)R_{j}^{kt}G_{j}^{kt}B_{j}^{kt};k=p,a$$

Then we can calculate the changed values $H_{ij}^{kt}S_{ij}^{kt}V_{ij}^{kt}$ of HSV between $n_{i}$ and the task projection ${n^{\prime }}_{j}$ (or task anchor $n_{j}$) by the equations below:

(14) $$H_{ij}^{kt}=H_{j}^{kt},S_{ij}^{kt}=S_{j}^{kt},V_{ij}^{kt}=(1-\frac{d_{ij}^{t}}{Range})\times V_{j}^{kt},k=p,a;d_{ij}^{t}=p_{ij}^{t}ord_{ij}^{t}=d_{t}(n_{i},n_{j})$$

where *Range* is the maximum length of color change, and we assume *Range* to be the maximum communication distance of the mobile node’s plane. Then $\left\{R_{ij}^{kt},G_{ij}^{kt},B_{ij}^{kt}\right\}$ between the mobile node $n_{i}$ and the task projection ${n^{\prime }}_{j}$ (or task anchor $n_{j}$) can be worked out based on the algorithm HSVtoRGB [12]:

(15) $$R_{ij}^{kt}G_{ij}^{kt}B_{ij}^{kt}=(HSV\text{ to }RGB)H_{ij}^{kt}S_{ij}^{kt}V_{ij}^{kt};k=p,a$$

At time instant t, the mobile node $n_{i}$ records the task anchors in $A_{i}^{t}$ and their depth information, then computes the distances from $n_{i}$ to the projections in $B_{i}^{t}$ for PCFL (or computes the distances from $n_{i}$ to the anchors in $A_{i}^{t}$ for ACFL), and $n_{i}$ processes normalization of these distances to calculate the proportion factor of distances weights $\lambda_{ij}^{t}$ defined as below:

(16) $$\lambda_{ij}^{t}=\frac{{\left(d_{ij}^{t}\right)}^{-1}}{\sum_{n_{j}\in A_{i}^{t}}{\left(d_{ij}^{t}\right)}^{-1}},d_{ij}^{t}=p_{ij}^{t}\;or\;d_{ij}^{t}=d^{t}(n_{i},n_{j})$$

At last using the weighted mean of $\left\{R_{ij}^{kt},G_{ij}^{kt},B_{ij}^{kt}\right\}$, RGB values $\left\{R_{i}^{Mt},G_{i}^{Mt},B_{i}^{Mt}\right\}$ for the mobile node $n_{i}$ can be worked out.

(17) $$\{R_{i}^{Mt},G_{i}^{Mt},B_{i}^{Mt}\}=\sum_{n_{j}\in A_{i}^{t}}\lambda_{ij}^{t}\{R_{ij}^{kt},G_{ij}^{kt},B_{ij}^{kt}\}\;k=p,a$$

The second stage is the RGB sequences calculation for samples. The RGB sequence for the sample $s_{k}$ is denoted by $RGB_{i}^{s_{k}t}:\{R_{i}^{s_{k}t},G_{i}^{s_{k}t},B_{i}^{s_{k}t}\}$. The calculation is similar to that mentioned above, and the difference just lies in replacing $d_{ij}^{t}$ in Equation (16) with the Euclidean distance between the mobile node $n_{i}$ and the sample $s_{k}$ which can be calculated using Equation (2). 

(c) In the process of filtering and weighted evaluation, PCFL and ACFL both filter the samples in the sampling area based on nearness degree. Assume at time instant t, PCFL and ACFL both sample m times randomly and the RGB sequence of the sample $s_{k}$ is $\left\{R_{i}^{s_{k}t},G_{i}^{s_{k}t},B_{i}^{s_{k}t}\right\}$, then the nearness degree $\mu_{s_{k}M}^{t}$ between the mobile node $n_{i}$ and the sample $s_{k}$ is defined as:

(18) $$\mu_{s_{k}M}^{t}=\sqrt{{\left(R_{i}^{s_{k}t}-R_{i}^{Mt}\right)}^{2}+{\left(G_{i}^{s_{k}t}-G_{i}^{Mt}\right)}^{2}+{\left(B_{i}^{s_{k}t}-B_{i}^{Mt}\right)}^{2}}(i=p+1,...q;k=1,2,...m)$$

Then the filtered samples set ${\tilde{S}}_{i}^{t}$ is:

(19) $${\tilde{S}}_{i}^{t}=\{s_{k}|s_{k}\in S_{i}^{t},\mu_{S_{k}M}^{t}\leq \mu^{t}\},n_{i}\in M$$

where $\mu^{t}$ is the threshold at time instant t*.* The relationship between $\mu^{t}$ and the localization error is shown in the simulation section. Assume there are $m^{t}(m^{t}\leq m)$ samples that have been filtered out. From Equations (18) and (19), the following Theorem 2 will prove the fact that the smaller the nearness degree $\mu_{s_{k}M}^{t}$ is the closer the mobile node $n_{i}$ is to the sample $s_{k}$, Then, based on $\mu_{s_{k}M}^{t}$, PCFL and ACFL perform the normalized weighted processing in the coordinate calculation of the mobile node $n_{i}$:

(20) $${\tilde{\mu }}_{S_{k}M}^{t}=\mu_{S_{k}M}^{t}{\left(\sum \mu_{S_{k}M}^{t}\right)}^{-1},s_{k}\in {\tilde{S}}_{i}^{t}$$

Assume the coordinate of the filtered sample $s_{k}$ is $\left(x_{s_{k}}^{t},y_{s_{k}}^{t},z_{i}^{t}\right)$, then the coordinate $\left(x_{i}^{t},y_{i}^{t},z_{i}^{t}\right)$ of the mobile node $n_{i}$ at time instant t can be calculated using ${\tilde{\mu }}_{s_{k}M}^{t}$ as the weights, note that $z_{i}^{t}$ can be achieved by the deployed pressure sensor:

(21) $$x_{i}^{t}=\sum_{S_{k}\in {\tilde{S}}_{i}^{t}}x_{S_{k}}^{t}{\tilde{\mu }}_{S_{k}M}^{t},y_{i}^{t}=\sum_{S_{k}\in {\tilde{S}}_{i}^{t}}y_{S_{k}}^{t}{\tilde{\mu }}_{S_{k}M}^{t}$$

The detailed procedure for PCFL and ACFL can be presented by the following Algorithm 1 description*.*

**Algorithm 1.** The PCFL and ACFL algorithms.1: **if** the mobile node $n_{i}$ senses the request command of localization **then**2: $n_{i}$ sends the acoustic signal in the communication range3: **if**
$n_{i}$ receives the acknowlegement signals from the task anchors **then**4: $n_{j}$ is named as the task anchor5: $\alpha_{ij}^{t}$ and $k_{ij}^{t}$ are measured by AOA and the deployed pressure sensors respectively6: $d^{t}(n_{i},n_{j})=k_{ij}^{t}/\sin\alpha_{ij}^{t}$7: $p_{ij}^{t}=k_{ij}^{t}/\tan\alpha_{ij}^{t}$8: **end if**9: **end if**10: **while**
$\left\{R_{j}^{at},G_{j}^{at},B_{j}^{at}\right\}$ is the RGB values for the task anchors$\left\{R_{j}^{pt},G_{j}^{pt},B_{j}^{pt}\right\}$ is the RGB values for the task anchors’ projections **do**11: $H_{j}^{kt}S_{j}^{kt}V_{j}^{kt}=(RGB\text{ to }HSV)R_{j}^{kt}G_{j}^{kt}B_{j}^{kt};k=p,a$12: $H_{ij}^{kt}=H_{j}^{kt},S_{ij}^{kt}=S_{j}^{kt},V_{ij}^{kt}=(1-\frac{d_{ij}^{t}}{Range})\times V_{j}^{kt},k=p,a;d_{ij}^{t}=p_{ij}^{t}ord_{ij}^{t}=d_{t}(n_{i},n_{j})$13: $R_{j}^{kt}G_{j}^{kt}B_{j}^{kt}=(HSV\text{ to }RGB)H_{j}^{kt}S_{j}^{kt}V_{j}^{kt};k=p,a$14: $\lambda_{ij}^{t}=\frac{{\left(d_{ij}^{t}\right)}^{-1}}{\sum_{n_{j}\in A_{i}^{t}}{\left(d_{ij}^{t}\right)}^{-1}},d_{ij}^{t}=p_{ij}^{t}\;or\;d_{ij}^{t}=d^{t}(n_{i},n_{j})$15: $\{R_{i}^{Mt},G_{i}^{Mt},B_{i}^{Mt}\}=\sum_{n_{j}\in A_{i}^{t}}\lambda_{ij}^{t}\{R_{ij}^{kt},G_{ij}^{kt},B_{ij}^{kt}\}\;k=p,a$16: **end while**17: **if**
$S_{i}^{t}=\{\left(x,y,z\right)|\forall {n^{\prime }}_{j}\in B_{i}^{t},\sqrt{\left(x-{x^{\prime }}_{j}\right)+{\left(y-{y^{\prime }}_{j}\right)}^{2}}\leq \sqrt{\left(R^{2}-{\left(k_{ij}^{t}\right)}^{2}\right)},z=z_{i}\}$
**then**18: $S_{i}^{t}$ is the set of the sampling area19: **end if**20: **if**
$\left\{R_{i}^{s_{k}t},G_{i}^{s_{k}t},B_{i}^{s_{k}t}\right\}$ is the RGB sequence of sample *s_k_*
**then**21: $\mu_{s_{k}M}^{t}=\sqrt{{\left(R_{i}^{s_{k}t}-R_{i}^{Mt}\right)}^{2}+{\left(G_{i}^{s_{k}t}-G_{i}^{Mt}\right)}^{2}+{\left(B_{i}^{s_{k}t}-B_{i}^{Mt}\right)}^{2}}(i=p+1,...q;k=1,2,...m)$22: ${\tilde{S}}_{i}^{t}=\{s_{k}|s_{k}\in S_{i}^{t},\mu_{S_{k}M}^{t}\leq \mu^{t}\},n_{i}\in M$23: ${\tilde{\mu }}_{S_{k}M}^{t}=\mu_{S_{k}M}^{t}{\left(\sum \mu_{S_{k}M}^{t}\right)}^{-1},S_{k}\in {\tilde{S}}_{i}^{t}$24: $x_{i}^{t}=\sum_{S_{k}\in {\tilde{S}}_{i}^{t}}x_{S_{k}}^{t}{\tilde{\mu }}_{S_{k}M}^{t},y_{i}^{t}=\sum_{S_{k}\in {\tilde{S}}_{i}^{t}}y_{S_{k}}^{t}{\tilde{\mu }}_{S_{k}M}^{t}$25: **end if**

From Algorithm 1, it can be seen that PCFL and ACFL are two localization methods for mobile nodes (line 1). In UASNs, nodes communicate with each other using acoustic signals (line 2). If the mobile node receives the feedback signals from the anchors within communication range, then, the anchors are named as the task anchors to localize the mobile nodes (lines 3, 4). When the mobile node obtains the feedback signals (including the depth information and the locations of the task anchors), the geographic distance $d^{t}(n_{i},n_{j})$ between the anchor $n_{i}$ and the mobile node $n_{i}$ can be obtained. At the same time, the distance $p_{ij}^{t}$ between the mobile node $n_{i}$ and the task anchor’s projection ${n^{\prime }}_{i}$ can be obtained too (lines 5, 6, 7). 

The RGB values for task anchors’ projections (PCFL) and task anchors (ACFL) are assigned (line 10). The HSV values for task anchors’ projections (PCFL) or for task anchors (ACFL) are calculated using the traditional convert algorithm RGB to HSV (line 11). Then the HSV values between the mobile node $n_{i}$ and the task projection ${n^{\prime }}_{j}$ (or task anchor $n_{j}$) can be calculated (line 12). The RGB values between the mobile node $n_{i}$ and the task projection ${n^{\prime }}_{j}$ (or task anchor $n_{j}$) can be calculated by the traditional convert algorithm HSVtoRGB (line 13). Then the proportion factor of distances weights $\lambda_{ij}^{t}$ can be calculated (line 14). The RGB values for the mobile node can be worked out using the weight means of the RGB values for the task projection ${n^{\prime }}_{j}$ (or task anchor $n_{j}$) (line 15). 

The sampling area can be determined based on the task-rings (line 17, 18). Then the RGB values for the samples are assigned (line 20). After that, the nearness degree $\mu_{s_{k}M}^{t}$ between the mobile node $n_{i}$ and sample $s_{k}$ can be calculated (line 21). If $\mu_{s_{k}M}^{t}$ is not more than the threshold $\mu^{t}$, the sample $s_{k}$ will be filtered out (line 22). Then PCFL and ACFL perform the normalized weighted processing (line 23). At last, the coordinate of the mobile node $n_{i}$ at time instant t can be calculated using ${\tilde{\mu }}_{s_{k}M}^{t}$ as the weights based on the coordinates of the filtered samples (line 24).

**Figure 4:**
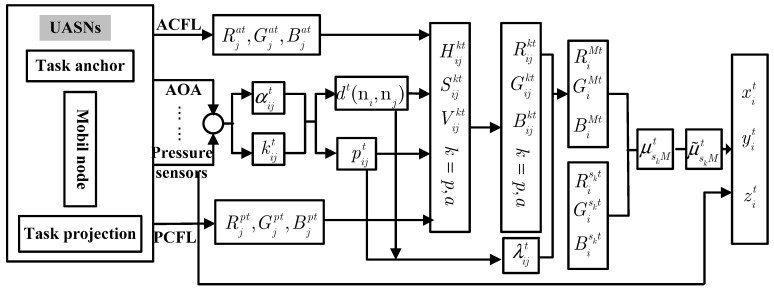
The architecture for PCFL and ACFL algorithm.

Figure 4 gives the architecture for both the PCFL algorithm and the ACFL algorithm.

### 3.3. Feasibility Analysis 

In this section, we analyze the feasibility of PCFL and ACFL, through the following theorems:

*Theorem 1*. Assume that $n_{i}$ is the mobile node, and ${n^{\prime }}_{j}$ is the task projection of task anchor $n_{j}$ $\left(n_{j}\in A_{i}^{t}\right)$ for $n_{i}$, that is, $d(n_{j},n_{i})\leq R$ and $x_{j}={x^{\prime }}_{j},y_{j}={y^{\prime }}_{j}$. Then, $n_{i}$ is inside the intersection area of the rings taking ${n^{\prime }}_{j}$ as the center and $\sqrt{R^{2}-{\left(k_{ij}^{t}\right)}^{2}}$ as the radius, correspondingly.

*Proof*. As for all $n_{j}\in A_{i}^{t}$, there is $d(n_{j},n_{i})\leq R$, we can see that $n_{i}$ is in the intersection area of the spheres taking $n_{j}$ as the centers and R as the radius, respectively, as shown in Figure 3. While, every sphere intersects the $n_{i}$ plane so as to get its ring. In view of these rings inside the $n_{j}$ plane would be the task-rings defined in Equation (7). Then, is in the intersection of these task-rings, such as one of them takes ${n^{\prime }}_{j}$ as the center and $\sqrt{R^{2}-{\left(k_{ij}^{t}\right)}^{2}}$ as the radius. The theorem can also verify correctness of the constraints in Equations (11) and (12) for the sampling area.

*Lemma 1*. Each RGB sequence is unique. 

*Proof.* The lemma can be proved by *redectio ad absurbum*. Suppose that in PCFL and ACFL there are two different mobile nodes (or samples) with the same RGB sequence at time instant t. This indicates that the two sets of corresponding task anchors are the same, and the two corresponding distances or corresponding projection distances are also same, although the mobile nodes (or samples) are different. However, this is a contradiction as, by the trilateration algorithm, two different nodes (or samples) in a three-dimensional region are the same when the distances between four anchors are known. 

*Theorem 2*.The smaller the nearness degree $\mu_{s_{k}M}^{t}$ is, the closer sample $s_{k}$ gets to mobile node $n_{i}$. 

*Proof*. It is clear that $\left\{R_{i}^{Mt},G_{i}^{Mt},B_{i}^{Mt}\right\}$ is unique to the mobile node $n_{i}$ and $\left\{R_{i}^{s_{k}t},G_{i}^{s_{k}t},B_{i}^{s_{k}t}\right\}$ is unique to the sample $s_{k}$ from Lemma 1. Consider two samples, represented as $s_{l}$ and $s_{k}$, respectively. Next, we assume that ${\tilde{d}}_{lj}^{t}({\tilde{d}}_{kj}^{t})$ denotes the distance between the sample $s_{l}$($s_{k}$) and the task projection ${n^{\prime }}_{j}$ (or task anchor $n_{j}$), respectively. Assume that:

(22) $$\mu_{s_{l}M}^{t}\leq \mu_{s_{k}M}^{t}$$

Then, the theorem can be proved by *redectio ad absurbum*. Suppose the sample $s_{k}$ is closer to the mobile node $n_{i}$ than the sample $s_{l}$ and:

(23) $$\left|{\tilde{d}}_{lj}^{t}-d_{ij}^{t}\right|<\left|{\tilde{d}}_{kj}^{t}-d_{ij}^{t}\right|,d_{ij}^{t}=p_{ij}^{t},(or\;d^{t}(n_{i},n_{j}))$$

So:

(24) $$\left|{\tilde{V}}_{lj}^{ft}-V_{ij}^{ft}\right|>\left|{\tilde{V}}_{kj}^{ft}-V_{ij}^{ft}\right|f=p,a$$

where ${\tilde{V}}_{lj}^{ft}({\tilde{V}}_{kj}^{ft})$ denotes the V value of HSV between the sample $s_{l}$($s_{k}$) and the task projection ${n^{\prime }}_{j}$ (or task anchor $n_{j}$). Then, by Equation (16):

(25) $$\left|{\tilde{\lambda }}_{lj}^{t}-\lambda_{ij}^{t}\right|>\left|{\tilde{\lambda }}_{kj}^{t}-\lambda_{ij}^{t}\right|$$

where ${\tilde{\lambda }}_{lj}^{t}({\tilde{\lambda }}_{kj}^{t})$ denotes the proportion factor of distances weights between the sample $s_{l}$($s_{k}$) and the task projection ${n^{\prime }}_{j}$ (or task anchor $n_{j}$).

By Equation (13) to Equation (15), we can see:

(26) $$\left|{\tilde{G}}_{lj}^{ft}-G_{ij}^{ft}\right|=\left|{\tilde{G}}_{kj}^{ft}-G_{ij}^{ft}\right|，\left|{\tilde{R}}_{lj}^{ft}-R_{ij}^{ft}\right|=\left|{\tilde{R}}_{kj}^{ft}-R_{ij}^{ft}\right|，\left|{\tilde{B}}_{lj}^{ft}-B_{ij}^{ft}\right|>\left|{\tilde{B}}_{kj}^{ft}-B_{ij}^{ft}\right|f=p,a$$

So by Equation (17), we have:

(27) $$\left|R_{i}^{s_{l}t}-R_{i}^{Mt}\right|>\left|R_{i}^{s_{k}t}-R_{i}^{Mt}\right|,\left|G_{i}^{s_{l}t}-G_{i}^{Mt}\right|>\left|G_{i}^{s_{k}t}-G_{i}^{Mt}\right|,\left|B_{i}^{s_{l}t}-B_{i}^{Mt}\right|>\left|B_{i}^{s_{k}t}-B_{i}^{Mt}\right|$$

Then:

(28) $$\mu_{s_{l}M}^{t}>\mu_{s_{k}M}^{t}$$

However, this is a contradiction to Equation (22). 

Next we will analysis the time and space consumption in the worst case for PCFL and ACFL.

*Theorem 3*. The new algorithms take O(*n*) worst case time and O(*n*) worst case space.

*Proof*. Let’s consider a 3D UASNs *UN* with *p* anchors as stated before, in the process (a) determination of the sampling area, it takes O(*p*) time for the mobile node to obtain the angles w.r.t. each task anchor using the AOA method and it requires O(*p*) space to store angles. Then it takes O(*p*) time to compute the distances from the mobile node to every task anchor according to the Equation (9) or to every task projection according to the Equation (10) and it requires O(*p*) space to store the depth information and O(*p*) space to store the distance. At last it takes O(*p*) time to confine the samples and it requires O(*p*) space to store these samples. 

In the process (b) RGB values calculation, it first takes O(*p*) time for the mobile node to convert the RGB values of the task anchors or of the task projections into HSV according to Equation (13) and it requires O(*p*) space to store these HSV values. Then it takes O(*p*) time to calculate the changed HSV values according to Equation (14) and it requires O(*p*) space to store the changed HSV values. Later it takes O(*p*) time to work out the changed RGB values according to Equation (15) and it requires O(*p*) space to store the changed RGB values. Finally it takes O(*p*) time to work out the proportion factor of distances weights and the RGB values for the mobile node according to Equations (16) and (17) and it requires O(*p*) space to store the proportion factor of distances weights and the RGB values for the mobile node.

In the process (c) filtering and weighted evaluation, assuming sampling *m* times randomly, then it takes O(*m*) time to work out the nearness degree according to Equation (18) and it requires O(*m*) space to store the nearness degree. It takes O(*m*) time to filter out the samples according to the Equation (19) and it requires O(*m*) space to store the filtered samples. At last it takes O(*m*) time to normalize weighted the nearness degree and calculate the coordinates of the mobile node according to Equations (20) and (21) and it requires O(*m*) space to store the normalized weighted nearness degree and the coordinates of the mobile node.

Therefore, in total, both PCFL and ACFL can be considered as taking O(*n*) time and O(*n*) space in the worst case to estimate the coordinates of the mobile node. 

Table 2 lists the comparison of worst case computational complexities between the typical algorithms and our new algorithms.

**Table 2:** Comparison of worst case time.

Algori-thms	PCFL/ACFL	Anchor-aid	AUV-aid	AFLA
Time	O(*n*)	O(*nlogn*)	O(*nlogn*)	O(*n*^2^)
Space	O(*n*)	O(*n*)	O(*n*)	O(*n*)

## 4. Simulation Results

In this section, we make a comprehensive evaluation for the PCFL algorithm and the ACFL algorithm through simulation experiments on Matlab 7.0. Three localization schemes, Anchor-based [20], AUV-aid [19] and AFLA [8] are compared with PCFL and ACFL. In the simulation experiments, the localization area is defined as 1000 m × 1000 m × 20 m where anchors are randomly deployed. The depth information of mobile nodes and anchors can be obtained by the pressure sensors at each time instant t. The original speed of mobile nodes which move in the ocean water is assumed to be 0.1 m/s. The maximum communication radius R is assumed to be 100 m. The localization error $E_{i}^{t}$ for the mobile node $n_{i},(i=p+1,...p+q),n_{i}\in M$ at time instant t can be calculated by [13]:

(29) $$E_{i}^{t}=\sum_{k=1}^{T}\sqrt{\left({\overset{⌢}{x}}_{i}^{t}-x_{i}^{t})+({\overset{⌢}{y}}_{i}^{t}-y_{i}^{t})+({\overset{⌢}{z}}_{i}^{t}-z_{i}^{t}\right)}/T$$

$\left({\overset{⌢}{x}}_{i}^{t},{\overset{⌢}{y}}_{i}^{t},{\overset{⌢}{z}}_{i}^{t}\right)$ and $\left(x_{i}^{t},y_{i}^{t},z_{i}^{t}\right)$ are the real coordinate and the estimated coordinate of the mobile node $n_{i}$, respectively. We run the simulation 50 times for each group of data, namely T=50.

Simulation parameters in this paper are summarized in Table 3.

**Table 3:** Simulation parameters.

Parameter	Value
Localization area	1000 m × 1000 m × 20 m
The maximum communication radius	100 m
The speed of mobile nodes	2 m/s–20 m/s
Speed of sound	1500 m/s
Error in speed of sound	0.07 m/s
The measurement error of communication angle	0°–10°
The number of samples	50–500
The density of anchors	0.5–5
The number of anchors	5–100
The number of deployed mobile nodes	20–100
Error in speed of sound	0.07 m/s
The times of simulation run	50

### 4.1. The Localization Error of ACFL and PCFL under Different Parameters

We compare localization performance of PCFL and ACFL through three different variables which are the threshold, the density of anchors and the number of samples.

#### 4.1.1. The Threshold ($\mu^{t}$)

**Figure 5:**
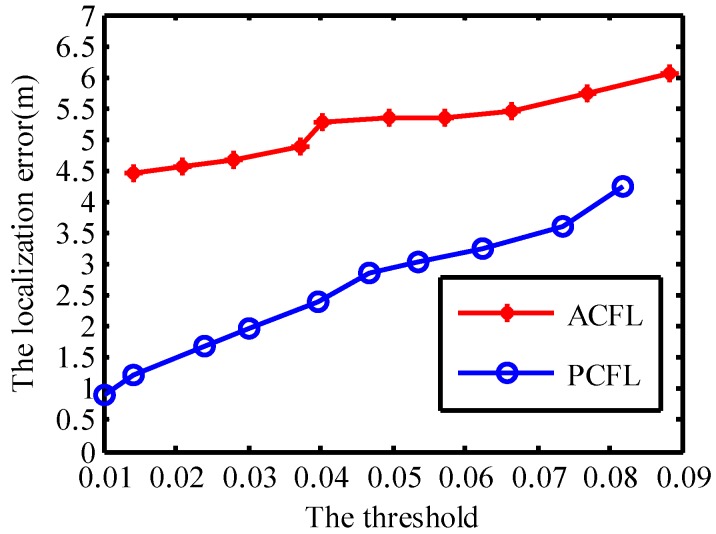
The threshold.

Figure 5 shows the localization error for both ACFL and PCFL changing with the threshold $\mu^{t}$. The density of anchors is set to be 4 and the number of samples is set to be 400 in Figure 5. In general, the larger the threshold, the less number the task anchors which can communicate with mobile nodes. The localization error of ACFL (PCFL) reaches the minimum, when the threshold is set for 0.0142 and 0.01, respectively. The localization error is increasing with the growth of the threshold. In general, the average localization error of ACFL is bigger than that of PCFL as shown in Figure 5. When the threshold is changed from 0.01 to 0.03, the average localization error of PCFL is less than 2 m, while that of ACFL is more than 4 m. 

#### 4.1.2. The Number of Samples 

**Figure 6:**
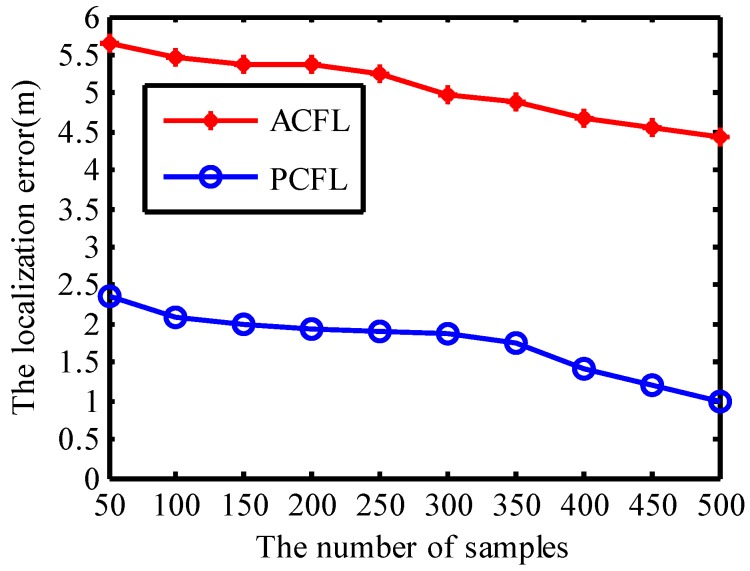
The number of samples.

Filtering out more samples for ACFL (PCFL) in the process of simulation experiments can improve the localization error, but consumes more energy. Considering this situation, the number of samples which are deployed to localize mobile nodes should be reasonable to reach a compromise between energy savings and localization accuracy. In Figure 6 the threshold is set to be 0.0142 and the density of anchors is set to be 4. 

When the number of the deployed samples which are filtered out to localize mobile nodes is much enough, samples are concentrated in the vicinity of the mobile node and the localization error reduces obviously. We can find that the localization error for both PCFL and ACFL is 0.91 m and 4.44 m when the number of the deployed samples is 500, respectively. 

#### 4.1.3. The Density of Anchors 

**Figure 7:**
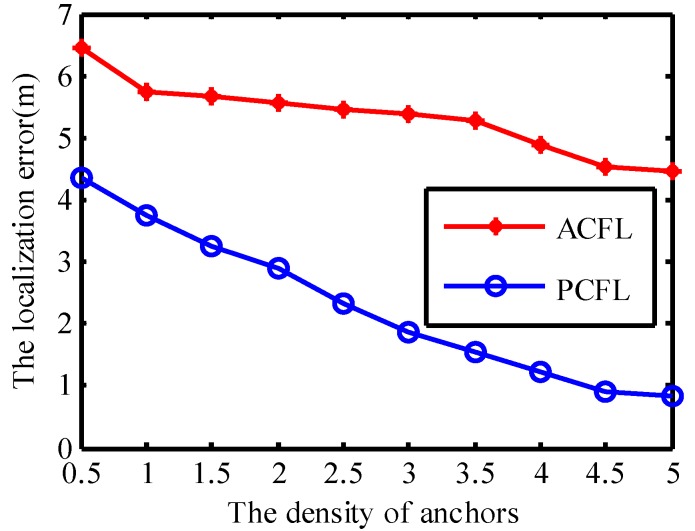
The density of anchors.

In Figure 7 the threshold is set to be 0.0142 and the number of samples is set to be 400. $N_{anchor}$ stands for the average number of task anchors in the communication radius *R* and is assumed to change from 5 to 100. We assume that V denotes the volume of the simulation space. $D_{anchor}$ is defined as the density of task anchors and it can be calculated by the formula $D_{anchor}=(\frac{4}{3}\pi R^{3})N_{anchor}/V$ [17], so we can obtain different values of the localization error when $D_{anchor}$ varies from 0.5 to 5 in the simulation experiment. We can filter out more task anchors with the increase of $D_{anchor}$. The localization error of PCFL (or ACFL) is inversely proportional to $D_{anchor}$ and decreases obviously along with the increase of $D_{anchor}$ as shown in Figure 7. Because PCFL (or ACFL) can convert three-dimensional localization algorithm to two-dimensional scenario, it can give accurate location and good reliability.

#### 4.1.4. The Estimated and Original Location 

**Figure 8:**
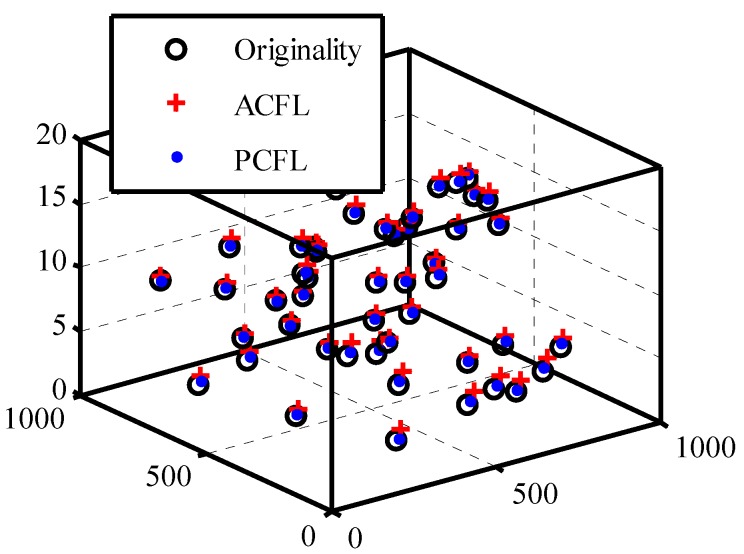
The original and estimated coordinates.

In the simulation space, 45 mobile nodes are randomly distributed to localize themselves, the distances between each pair of them are 120 m and 160 m. As we can see in Figure 8, black circles represent the original position, red and blue signs are the estimated coordinates computed by ACFL and PCFL, respectively, so it is quite clear that calculations of PCFL are more close to actual locations. 

We can find that localization error of PCFL (or ACFL) reduces slightly when the number of samples is more than 400. When $D_{anchor}$ is 4 or larger, the localization error varies smoothly. Based on the running time of simulation experiments and localization error, we set the threshold for 0.0142, $D_{anchor}$ for 4, and the number of samples for 400 in the following simulation experiments.

#### 4.1.5. Total Energy Consumption

**Figure 9:**
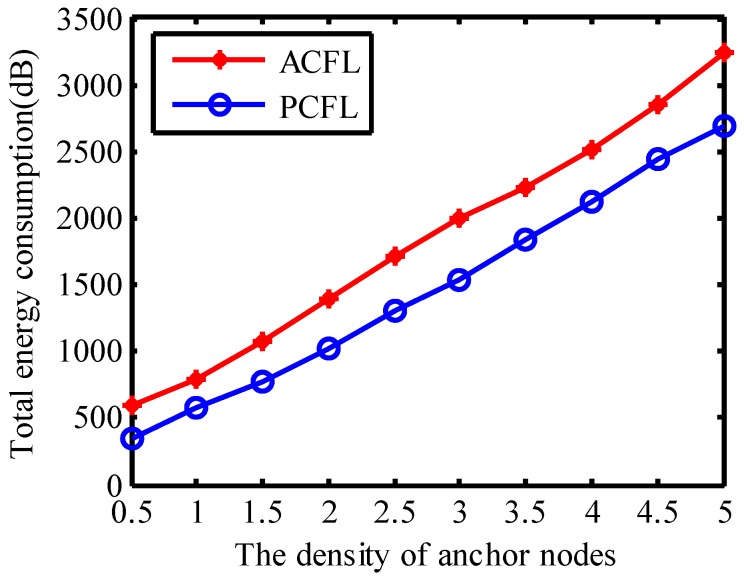
Total energy consumption.

In Figure 9 the threshold is set to 0.0142 and the number of samples is set to 400. The energy consumption which is based on the energy consumption model referred by [22,23] can be expressed by:

(30) $$\alpha (f)=\frac{0.1f^{2}}{1+f^{2}}+\frac{40f^{2}}{4100+f^{2}}+2.75\times 10^{-4}f^{2}+0.003$$

(31) $$E=p_{0}\times d_{ij}^{t}\times 10^{(\alpha (f))/10};d_{ij}^{t}=p_{ij}^{t}ord_{ij}^{t}=d^{t}(n_{i},n_{j})$$

where $p_{0}$ as a constant is the least energy consumption level. f denotes the frequency and its unit is Hz, α(f) represents the absorption coefficient relying on the frequency value, underwater temperature and underwater salinity, its unit is dB/km. and $d_{ij}^{t}$ denotes the distance between the mobile node and the anchor (or the anchor’ projection).

It is obvious that total energy consumption for either PCFL or ACFL is increasing with the density of anchors. Energy consumption for ACFL is more than that for PCFL. This also verifies the high energy efficiency of PCFL.

#### 4.1.6. Runtime 

**Figure 10:**
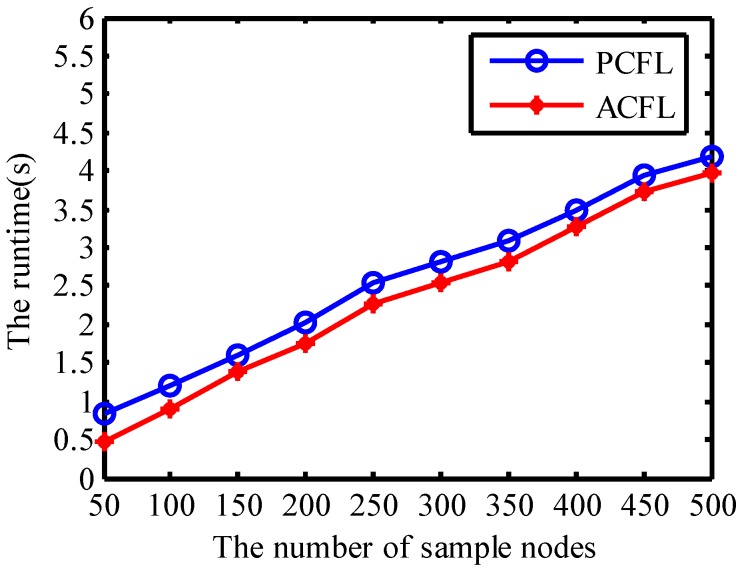
Runtime.

In Figure 10 the threshold is set to 0.0142 and the density of anchors is set to 4. Figure 10 presents the simulation experiment runtime using PCFL and ACFL. Since the runtime is the process of data association in fact, the runtime for either PCFL or ACFL is increasing with the growth of the number of the samples. Because the operation process of PCFL is more complicated than that of ACFL, the runtime of ACFL is shorter than that of PCFL.

### 4.2. Comparison with Different Methods

Here, we run the simulation 50 times for each localization error in Equation (29), then the average localization error can be obtained. We compare the five algorithms as shown in Table 4 which lists the maximum localization error, the minimum localization error, the average localization error and the standard deviation of the five algorithms. 

**Table 4:** Comparison of localization errors.

Algorithm	Average Error(m)	Max Error(m)	Min Error(m)	Standard Deviation(m)
Anchor-based	5.59	16.86	2.81	4.06
AUV-aid	9.82	7.72	1.56	2.69
AFLA	2.63	13.89	0.31	1.8
ACFL	4.56	10.22	0.51	2.06
PCFL	1.83	5.01	0.14	0.87

The localization error of the Anchor-based algorithm is susceptible to the error of the estimated distances in relation to the coordinates of the fixed anchors, so the maximum localization error, the minimum localization error and the standard deviation of the Anchor-based algorithm are larger than the other four algorithms. Utilizing geometrical relationships, the AUV-aid method performs localization coarsely, so its average localization error is the biggest. We can see that the average localization error of the ACFL is 4.56, which is larger than that of the AFLA and the PCFL according to the simulation results. Table 4 shows that the PCFL algorithm has better localization performance and smaller localization error, compared with the other four algorithms. The average localization error of PCFL can decline by about 30.4% than AFLA method. 

### 4.3. The Percentage Distribution of the Localization Error

**Figure 11:**
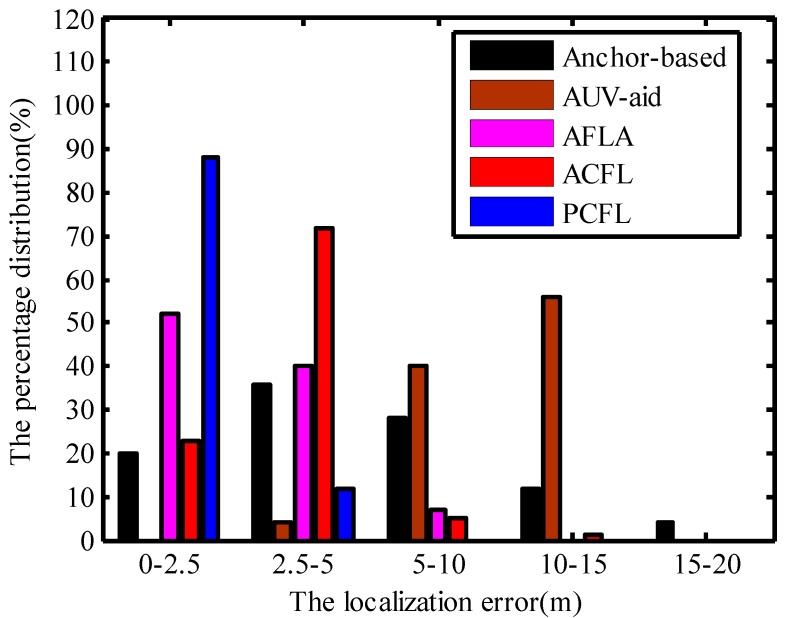
The percentage distribution.

Figure 11 shows the distribution histogram of the localization error, comparing the Anchor-based algorithm, the AUV-aid algorithm, the AFLA algorithm, the ACFL algorithm and the PCFL algorithm. We deploy 20 mobile nodes stochastically in the simulation region. All mobile nodes perform self-localization simultaneously by running the experiment 50 times independently. When the localization error of PCFL and the localization error of ACFL are both less than 2.5 m, the percentage distribution is 88% and 23%, respectively. However the distribution percentage of the localization error of AFLA is lower than that of PCFL. And the distribution percentage of the localization error of Anchor-based is also lower than that of PCFL when the range of the localization error is from 0 to 2.5. The percentage distribution of the localization error of AFLA is the highest compared with the other four algorithms when the localization error is varying from 2.5 to 5. The percentage distribution of PCFL is zero when the localization error is varying from 5 to 20. In a word, PCFL has better localization performance and smaller localization error.

### 4.4. Error and the Speed of Mobile Nodes

**Figure 12:**
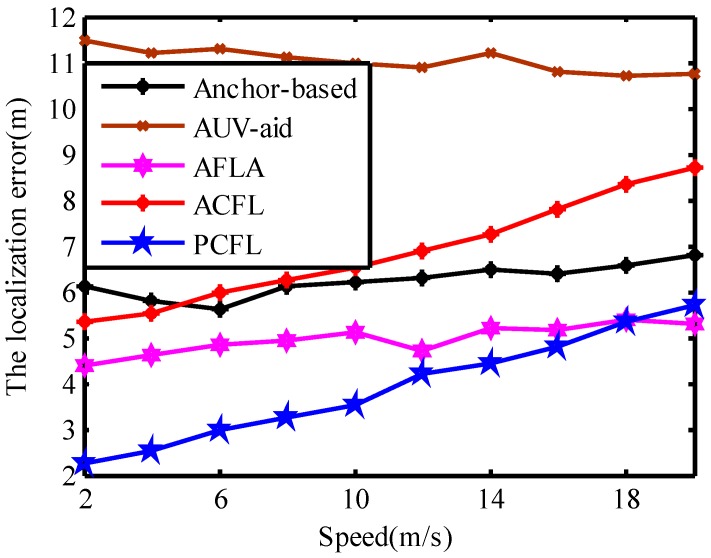
The speed of mobile nodes.

The speed of mobile nodes is an important factor affecting the localization error. Here the speed of mobile nodes is set to vary from 2 m/s to 20 m/s. The results of the simulation experiments as shown in Figure 12 indicate that the faster the moving speed, the bigger the localization error is. Due to using geometrical relationship to localize the mobile nodes coarsely, the localization error of AUV-aid is the maximum and changes stable without downtrend. AFLA used the geographical relationship of neighbor nodes to localize mobile nodes which did not need the information of anchors, so its precision is affected slightly by the speed of mobile node. Since the faster mobile nodes move, the less number of task anchors which can contact with them in three dimensional UASN can be acquired. So with the speed of mobile nodes increasing, the localization error of PCFL is almost equal to that of AFLA. And the overall varying trend of the localization error of PCFL or ACFL rises faster than the other three algorithms. When the speed is lower than 18 m/s, the localization error of PCFL is the minimum. As a whole, PCFL exceeds the other four methods with its average localization error of 3.92 m, much smaller than that of ACFL, 7.33 m, due to the projection technology.

### 4.5. Error and the Number of Deployed Mobile Nodes

**Figure 13:**
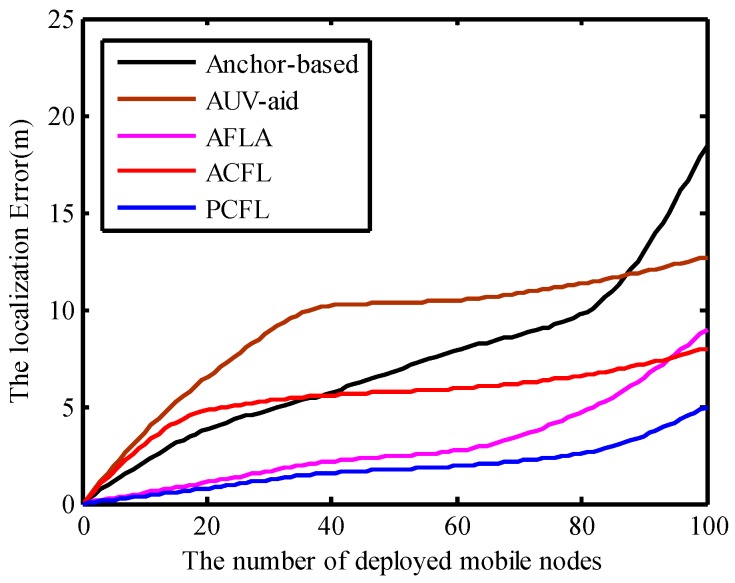
The number of deployed mobile nodes.

One hundred mobile nodes whose locations are unknown are randomly deployed in the three dimensional localization region. As we can see, in the process of simulation experiments, the five algorithms could localize all the deployed mobile nodes. Because the angles between task anchors and mobile nodes are calculated using the AOA method, the estimated distances between them have low accuracy and the localization error rises obviously with the number of the deployed mobile nodes increasing. The localization error of PCFL is smaller than that of the other four methods in Figure 13.

The average localization error of AUV-aid is bigger than the other four methods. The error variation of the Anchor-based method fluctuates clearly and reaches the maximum value. Compared with ACFL, PCFL is an effective self-localization algorithm, its localization error changes stable and keeps the smallest in the localization process.

## 5. Conclusions

In this paper, two algorithms called PCFL and ACFL using the color filtering method have been proposed for mobile node self-localization in UASNs. The PCFL method is based on the RGB values of task anchors’ projections, while ACFL is based on the RGB values of task anchors. The two methods can improve the coarse localization by combining the CDL method with the AOA measurements. Also the proportion factor of distance can optimize the CDL method and the nearness degrees can help filtering samples more precisely. The proposed methods present better accuracy and robustness than the Anchor-aid, AUV-aid and AFLA methods, especially the PCFL method, when the speed of the mobile nodes is lower. We plan to expand this work with actual underwater experiments and reduce the localization computational complexity in the future. 
